# Maternal Diet Quality and Prenatal Depressive Symptoms: The Moderating Role of Economic Well-Being

**DOI:** 10.3390/nu15122809

**Published:** 2023-06-20

**Authors:** Peiyi Wang, Ilona S. Yim, Karen L. Lindsay

**Affiliations:** 1 Department of Psychological Science, University of California, Irvine, CA 92617, USA; peiyi.wang@uci.edu (P.W.); ilona.yim@uci.edu (I.S.Y.); 2 Department of Pediatrics, School of Medicine, University of California, Irvine, CA 92617, USA; 3 UCI Susan Samueli Integrative Health Institute, College of Health Sciences, Irvine, CA 92617, USA

**Keywords:** diet quality, depressive symptoms, economic well-being, income, poverty, nutrition, pregnancy, prenatal

## Abstract

Prenatal depression is prevalent and adversely impacts maternal and infant health. This study addresses a critical literature gap and investigates the association between maternal diet quality and prenatal depressive symptoms, as well as the moderating effect of economic well-being on this link. A cross-sectional design was used, including 43 healthy pregnant women in the second trimester aggregated from two research projects. Prenatal depressive symptoms were assessed using the Edinburgh Postnatal Depression Scale. Dietary quality was evaluated using two non-consecutive 24 h dietary recalls, from which the Adapted Dietary Inflammatory Index (ADII) and the Healthy Eating Index (HEI)-2015 were derived. Economic well-being was indicated by the income-to-poverty ratio. A higher HEI-2015 (adherence to dietary guidelines; *β* = −0.53, *p* = 0.01) and negative ADII (anti-inflammatory diet; *β* = 0.40, *p* = 0.06) were associated with fewer prenatal depressive symp-toms. Among pregnant women with worse economic well-being, a pro-inflammatory diet was as-sociated with more prenatal depressive symptoms (*b* = 1.69, *p* = 0.004), but among those with better economic well-being, the association was not significant (*b* = 0.51, *p* = 0.09). Dietary interventions aimed at reducing dietary inflammation might hold some promise for improving mental health among pregnant women who are economically vulnerable.

## 1. Introduction

Nutrition is an essential determinant of maternal health during pregnancy and exerts a significant influence on fetal development and offspring health [1,2,3,4]. Prenatal depression is prevalent, particularly during the second trimester [5], which can lead to postpartum depression and poor child health outcomes, such as low birth weight and various emotional, behavioral, and neurocognitive problems in later developmental stages [6,7,8]. The effect of nutrition and diet on depression has been examined in non-pregnant populations, such that an overall healthy dietary pattern with high intakes of fruit, vegetables, fish, and whole grains is related to a reduced risk of depression [9,10].

However, studies investigating the link between maternal diet quality and prenatal depression suffer from inconsistencies and methodological biases [11,12,13]. Specifically, some research on nutrition and depression during pregnancy has only examined a single nutrient or used a single dietary recall, which may inadequately capture the intricate interplay between nutrition and mental health [12]. For example, some review papers conclude that specific nutrients with anti-inflammatory properties, such as omega-3 polyunsaturated fatty acids indicated by dietary consumption and plasma biomarkers [14,15], may be associated with fewer prenatal depressive symptoms. However, other studies failed to find relevant links between single nutrient intakes and prenatal depression [12,16,17,18]. Furthermore, though certain demographic factors have been posited to impact the relationship between nutrition and depression, the exact nature of their effects remains elusive [11]. Given that diet is a modifiable behavior with potential long-term health benefits, a more comprehensive understanding of the link between maternal diet quality and prenatal depression, as well as the factors influencing this association, is imperative. Such knowledge may facilitate the development of tailored nutritional interventions to support maternal mental health during and after pregnancy, as well as fetal and neonatal neurodevelopmental outcomes.

Aside from individual nutrients, overall diet quality may have a greater impact on the development of health outcomes through the potential synergistic effects of an array of nutrients and other food components, such as fiber and polyphenolic compounds [19,20]. Several dietary indices have been established to assess overall diet quality, including the Adapted Dietary Inflammation Index (ADII) and the Healthy Eating Index (HEI), which have been used in prenatal nutrition research [21,22,23]. Though the ADII is distinctive in its ability to identify the inflammatory potential of the overall diet [24], it is less frequently utilized in pregnancy and depression research compared to the HEI, which gauges adherence to dietary guidelines intended for the general adult population in the US. Nevertheless, given the established association between inflammation and depression [25], a diet of pro-inflammatory potential might also influence prenatal depressive symptoms and thus merit further investigation. Investigating the association between overall diet quality and prenatal depression using these two dietary indicators could advance our understanding of which features of the diet relate to maternal mental health outcomes, thereby improving the efficiency of nutrition assessment for future research in this field.

Economic well-being is a significant demographic factor that may moderate the association between maternal diet quality and prenatal depressive symptoms. Economic well-being characterizes the state of an individual’s economic health, which can be assessed using indicators such as income and the number of dependents relying on that income [26]. Poor economic well-being might represent a contextual factor that causes greater depression among the general population [27,28,29] and pregnant women [30,31]. Specifically, pregnant women who face unfavorable economic circumstances often worry about their ability to care for their children, experience increased stress, encounter employment instability, and may face social isolation due to their role as homemakers, which are risk factors for prenatal depression [31]. Importantly, pregnant women who experience food insecurity [32] and reside in nutritionally deficient areas [33] due to economic challenges may encounter a range of biopsychosocial adversities that can contribute to a downward spiral from poor dietary quality to depression [34]. Several systematic reviews have suggested that economic status may moderate the relationship between other risk factors and depression during pregnancy [35,36,37]. When pregnant women experience poor economic well-being, the magnitude of the association between maternal diet and prenatal depression might be stronger than in those who have better economic well-being.

The first aim of the present study was to examine the relationship between maternal diet quality and prenatal depressive symptoms in healthy pregnant women, using both the ADII and HEI-2015 to characterize overall diet quality. We hypothesized that a lower quality maternal diet, and in particular, a diet of greater pro-inflammatory potential, would be associated with more prenatal depressive symptoms. The second aim was to explore whether economic well-being would moderate the association between maternal diet quality and prenatal depressive symptoms. It was hypothesized that the strength of the relationship between poor diet quality and more prenatal depressive symptoms would be greater among pregnant women with lower economic well-being compared to those with higher economic well-being.

## 2. Materials and Methods

### 2.1. Study Procedures

Data for the current study were derived from two distinct research projects involving healthy pregnant women at the University of California, Irvine (UCI). The first project was a pilot feasibility study of a mindfulness-based intervention in pregnancy. The second project was a crossover study investigating the postprandial metabolic response to a meal following exposure to acute psychological stress. This paper utilized baseline dietary and psychological data from each project before interventions were individually administered.

Recruitment for both projects took place between June 2021 and February 2022. Both studies recruited participants attending prenatal care at obstetric clinics affiliated with the UCI Medical Center in Orange County, California, USA. Although the studies had different primary research objectives, they both utilized similar eligibility criteria (see below) and measured the variables of relevance to the current investigation at baseline, before any interventions were administered. Participants were compensated with a modest monetary incentive in both studies. The UCI institutional review board approved the two studies, and all participants provided written informed consent.

Participants completed a socio-demographic survey and psychological assessments via REDCap (i.e., an online data acquisition platform) during Zoom visits with the research coordinator. The sociodemographic survey included questions about participants’ age, ethnicity (Hispanic/non-Hispanic), total family income, and number of dependents on the income.

An initial interviewer-led 24 h diet recall was completed using the Automated Self-Administered—24 h (ASA24) online tool from the National Cancer Institute. The ASA24 is a validated, web-based dietary assessment instrument that provides nutrient analysis of all foods and beverages reported throughout the period of data collection. To capture within-individual day-to-day variation in diet, a second interviewer-led dietary recall was conducted on a non-consecutive day within one week of the initial diet recall, following past practices [38] and recommendations [39,40,41]. The dietary intake data from the two measurements were averaged to minimize potential measurement errors and enhance the validity of the data [40]. Using ASA24 as a method is considered advantageous due to its cost-effectiveness and less burdensome nature for the participants, which can potentially increase participation rates [42]. The ASA24 output generates values for daily food, calorie, and nutrient intake, which were scrutinized to identify potentially unrealistic values. Caloric intake below 800 kcal or exceeding 4400 kcal is usually considered implausible, and no such implausible calorie intake was observed in the current data. All survey and diet recall responses were recorded under de-identified study ID numbers.

### 2.2. Participants

Participants from the two parent studies who completed two dietary recalls were eligible for inclusion in the current research. Except for two individuals who reported only one dietary recall, all participants from the first study were included. Four individuals from the second study failed to report two dietary recalls at the time of data analysis (February 2022) and were therefore excluded from this research.

Thus, data from 43 pregnant women were included in the current analysis. In both studies, women were eligible to participate if they were at least 18 years old, nondiabetic, nonsmokers (either prior to or during the index pregnancy), fluent in either English or Spanish, carrying a singleton, having an intrauterine pregnancy, and were between 77 and 161 days of gestational age (GA) at the time of enrollment. GA was confirmed by the estimated date of delivery based on the medical records of the ultrasound result for most participants and was self-reported when medical record access was unavailable. Individuals were excluded from both studies if they had major obstetric complications or conditions that may dysregulate neuroendocrine, metabolic, or cardiovascular function, used corticosteroid or psychotropic medication, were under diagnosis or treatment of psychiatric disorders (e.g., depression), or used systemic/frequent corticosteroids or thyroid, lipid-lowering, or anti-diabetic medications. Additionally, if current substance abuse was documented in the electronic medical record when screening patients for recruitment purposes, those individuals were not invited to participate in the research. In Study 1 (*n* = 17), there was no restriction on pre-pregnancy body mass index (BMI), whereas Study 2 (*n* = 26) recruited participants whose pre-pregnancy BMI was in the 25.0–39.9 kg/m^2^ range. BMI was computed using self-reported pre-pregnancy weight and height with verification of these values from the medical record, where available.

### 2.3. Measures

#### 2.3.1. Adapted Dietary Inflammatory Index (ADII)

The ADII indicates the inflammatory potential of one’s diet [24]. In the present study, the ADII was calculated as previously described using the dietary recall data [24,43]. Twenty-five nutrient parameters were used to compute the index: protein, saturated fat, monounsaturated fat, omega-3 fatty acids, omega-6 fatty acids, cholesterol, carbohydrates, dietary fiber, alcohol, vitamins A (retinol equivalents), B1, B2, B3 (niacin), B6, B9 (folate from food), B12, C, E, and D, beta-carotene, iron, magnesium, zinc, selenium, and caffeine. Food and nutrient intakes across the reporting days were first averaged for each participant. Next, the individual food/nutrient components of the ADII were converted to z-scores by subtracting the global-standardized mean value from each variable and dividing it by its global standard deviation value. The z-scores for nutritional parameters were then multiplied by their respective inflammatory effect scores to obtain the nutrient parameter-specific ADII score. The total ADII score was determined by summing the scores for each parameter. A diet with a positive ADII score is considered pro-inflammatory, whereas one with a negative score is anti-inflammatory.

#### 2.3.2. Healthy Eating Index-2015 (HEI-2015)

The HEI-2015 evaluates compliance with the 2015–2020 Dietary Guidelines for Americans. This index contains 13 food groups, with nine adequacy components and four moderation components. The adequacy components should be consumed in adequate quantities to provide the necessary nutrients for overall health, whereas the moderation components should be consumed in limited amounts. The nine adequacy components include total fruits, whole fruits, total vegetables, greens and beans, whole grains, dairy, total protein foods, seafood and plant proteins, and fatty acids. The four moderation components are composed of refined grains, sodium, added sugars, and saturated fats. The dietary recall data supplies the food categories and other dietary components necessary to compute the HEI-2015 using the simple scoring algorithm method, as previously described [44]. The total HEI-2015 score was calculated by first averaging all the relevant food and nutrient intakes across the reporting days for each participant, then running the algorithm using averaged values for individual components. The total maximum score for the HEI-2015 is 100, and higher values indicate closer dietary guideline adherence and better diet quality.

#### 2.3.3. Economic Well-Being

The income-to-poverty ratio was used to assess respondents’ economic well-being. To calculate the ratio, the total self-reported family income was divided by the 2021 Federal Poverty Guidelines for the 48 contiguous states and the District of Columbia, according to the number of dependents on the income [45,46]. A higher income-to-poverty ratio signifies a more substantial deviation from the poverty threshold and thus better economic well-being.

#### 2.3.4. Prenatal Depressive Symptoms

Prenatal depressive symptoms were measured using the 10-item Edinburgh Postnatal Depression Scale (EPDS), a widely used self-report scale that has been validated for use during pregnancy and postpartum [47]. Respondents rated how often in the past 7 days they had experienced the described thoughts or feelings on a 4-point Likert-type scale, ranging from 0 = “never” to 3 = “very often”, with possible total values ranging from 0 to 30. Higher scores suggested more prenatal depressive symptoms. The Cronbach’s alpha level of this scale in the current study was 0.82.

### 2.4. Statistical Analysis

All analyses were performed using SPSS, version 24.0. Given the scarcity of existing research on the specific topic of interest, conducting an a priori power analysis was not feasible due to the unavailability of effect size information [48]. Moreover, in line with recommended practices, we refrained from conducting a post hoc power analysis, as it is widely acknowledged as inappropriate and potentially misleading [49,50,51,52]. Instead, confidence intervals have been reported for all the results, thus offering a more robust and informative interpretation of the findings [50].

Descriptive statistics were used to present participant characteristics (i.e., age, GA, pre-pregnancy BMI, and ethnicity), dietary quality, economic well-being, and prenatal depressive symptoms in the total cohort. Mean differences in these variables by study enrollment and ethnicity were assessed using the independent sample *t*-test. Pearson correlations were used to test the bivariate association between age, GA, pre-pregnancy BMI, economic well-being, dietary indices, and the EPDS score. Correlations were deemed as significant at the 0.05 significance level and as marginally significant at the 0.10 significance level. 

Two hierarchical regression models were conducted to test whether the two dietary indices were respectively associated with the prenatal depressive symptoms score and whether economic well-being was a moderator in these associations. Because age, GA, and prepregnant BMI are theoretically closely related to the onset and symptoms of prenatal depression [53], these three variables were the planned covariates. Sensitivity analysis was also conducted with and without the study enrollment covariate. In both models predicting the EPDS score, age, GA, and pre-pregnancy BMI were included as planned covariates in Step 1. In the first model, the ADII score and economic well-being were mean-centered and entered as the predictor variables in Step 2, and the interaction term (i.e., the mean-centered ADII score × economic well-being) was included in Step 3. In the second model, the HEI-2015 score and economic well-being were mean-centered and entered as the predictor variables in Step 2, and the interaction term (i.e., the mean-centered HEI-2015 score × economic well-being) was included in Step 3. The simple slope test and Johnson-Neyman procedure were carried out in the significant moderation result using PROCESS Macro (version 3.4) with 5000 bootstrapped resamples.

## 3. Results

### 3.1. Sample Characteristics

Descriptive statistics for the overall sample and stratified by study enrollment and ethnicity are presented in Table 1. Participants’ age ranged between 18 and 41 years old. At the time of study enrollment, the mean GA was 130.70 days (range, 74–158 days) and pre-pregnancy BMI was 31.07 kg/m^2^ (range, 22.31–42.06 kg/m^2^). Most participants identified as Hispanic (72.1%) compared to non-Hispanic (27.9%). Participants reported relatively low levels of prenatal depressive symptoms (*M* = 7.07, *SD* = 4.19; range, 0–15), a score that fell below the typically used cut-off score (*M* ≥ 13) for probable major depression in pregnancy [54]. The mean ADII score (*M* = 0.03, *SD* = 2.91; range, −7.19–6.17) reflected a diet of marginal pro-inflammatory potential [24]. The mean HEI-2015 total score (*M* = 60.63, *SD* = 13.37; range, 29.08–86.77) suggested a moderate quality diet in this sample, although it was less than the average score reported among pregnant women in the US (*M* = 63) [55].

Participants from Study 1 were earlier in GA at enrollment compared to those from Study 2 (*p* = 0.002). There were no other mean differences in demographic characteristics and main study variables based on study participation (all *p*s > 0.38). Hispanic women tended to be younger in age (*p* = 0.004) and have worse economic well-being (*p* = 0.02) compared to non-Hispanic pregnant women. No other mean difference in demographic characteristics and main study variables based on ethnicity was significant (all *p*s > 0.09).

The bivariate correlation coefficients of demographic characteristics and the main study variables are also presented in Table 2. A negative ADII score and a higher HEI-2015 score were associated with lower EPDS scores, suggesting that pregnant women who have a more anti-inflammatory diet and follow dietary guidelines more closely tend to have fewer prenatal depressive symptoms. A negative ADII score and a positive HEI-2015 score were also associated with older age, suggesting that pregnant women who are older tend to have a more anti-inflammatory diet and follow dietary guidelines more closely. Economic well-being was also associated with age, such that pregnant women who are older tend to have better economic well-being. Prenatal depressive symptoms were not significantly related to any demographic variables.

### 3.2. ADII, Economic Well-Being, and Prenatal Depressive Symptoms

The ADII score was marginally positively correlated with prenatal depressive symptoms (Table 3, Model 1), suggesting that a diet with pro-inflammatory potential was associated with more prenatal depressive symptoms on a trend level. The model was significantly improved when the interaction term (the mean-centered total ADII score × economic well-being) was added in Step 3, and the overall model explained a significant 36% variation in the EPDS score.

**Figure 1:**
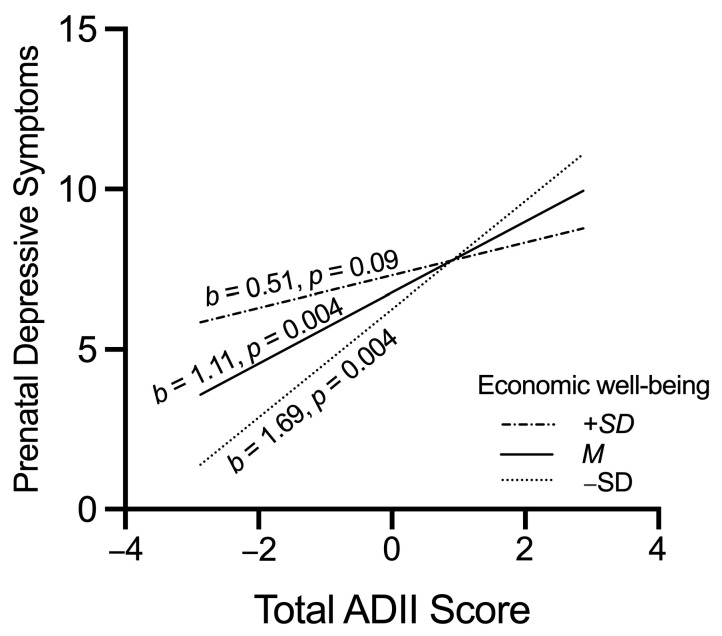
Moderation effect of economic well-being in the association between the Adapted Dietary Inflammatory Index (ADII) score and prenatal depressive symptoms. The dashed-dotted line indicates that the relationship between ADII and EPDS scores was nonsignificant when economic well-being was one standard deviation above the average value. The solid line indicates that the relationship between higher ADII and higher EPDS scores was significant when economic well-being was at the mean value. The dotted line indicates that the relationship between higher ADII and higher EPDS scores was significant when economic well-being was one standard deviation below the average value.

Among pregnant women who reported better economic well-being, the association between ADII and EPDS scores was not statistically significant (*b* = 0.51, *p* = 0.09, 95% CI_boot_ [−0.08, 1.10]). Comparatively, among pregnant women who have worse economic well-being, a pro-inflammatory diet was associated with more prenatal depressive symptoms (average: *b* = 1.11, *p* = 0.004, 95% CI_boot_ [0.38, 1.84]; low: *b* = 1.69, *p* = 0.004, 95% CI_boot_ [0.59, 2.79]; Figure 1). Thus, among pregnant women with economic well-being levels one standard deviation above the sample average, there was no significant association between the ADII score (i.e., a more pro-inflammatory diet) and EPDS score (i.e., prenatal depressive symptoms). In contrast, among pregnant women with economic well-being levels at the sample average level or one standard deviation below the sample average, each unit increase in the ADII score (i.e., a more pro-inflammatory diet) corresponded to a 1.11 or a 1.69 unit increase in EPDS score (i.e., prenatal depressive symptoms).

A similar moderation effect was observed using the Johnson-Neyman procedure. A significant link between a pro-inflammatory diet and more prenatal depressive symptoms (*b* ranged from 0.59 to 1.69) appeared at values below an economic well-being score of 2.93 (82.35% of the sample). A correlation between ADII and EPDS scores was not significant when the economic well-being score was equal to or above 2.93 (17.65% of the sample). The results held when the study variable was included in the model (interaction term = −0.18, beta = −0.52, *p* = 0.03, 95% CI = −0.33, −0.02).

Thus, the statistical tests collectively indicated that economic well-being moderated the strength of the relationship between a pro-inflammatory diet and prenatal depressive symptoms.

### 3.3. HEI-2015, Economic Well-Being, and Prenatal Depressive Symptoms

The HEI-2015 score showed a main effect on prenatal depressive symptoms (Table 3, Model 2), suggesting that a diet following more closely to dietary guidelines was associated with fewer prenatal depressive symptoms. This association was not moderated by economic well-being. Thus, among pregnant women in this study, each unit increase in the HEI-2015 score (indicating compliance with dietary guidelines) corresponded to a 0.18 unit decrease in EPDS score (reflecting prenatal depressive symptoms). This main effect was consistent irrespective of women’s economic well-being. The main effect of HEI-2015 on prenatal depressive symptoms was consistent when the study variable was included in the model (main effect = 0.19, beta = −0.57, *p* = 0.01, 95% CI = −0.32, −0.06).

## 4. Discussion

Consistent with our prediction, an overall higher-quality maternal diet characterized by an anti-inflammatory potential and a greater adherence to dietary recommendations was associated with fewer prenatal depressive symptoms. Furthermore, economic well-being moderated the association between diet quality and prenatal depressive symptoms when the maternal diet was measured by the ADII, but not the HEI-2015. Pregnant women experiencing worse (vs. better) economic well-being may exhibit increased vulnerability to prenatal depressive symptoms when adhering to a pro-inflammatory dietary pattern (e.g., a diet rich in saturated fat and sugar and with low consumption of fiber and micronutrient-rich foods). This study extends research on the connection between maternal nutrition and mental health outcomes during pregnancy and is among the first to examine a probable demographic moderator in the association.

Existing studies on nutrition and prenatal mental health [56] support the association between a higher-quality maternal diet and fewer prenatal depressive symptoms. When a suboptimal-quality diet is consumed, it can contribute to maternal nutritional depletion, which has been linked to poor mental health outcomes [57]. During pregnancy, there is an increased demand for nutrients due to the developing fetus, but inadequate intake of omega-3 polyunsaturated fatty acids, folate, B vitamins, iron, and calcium are common and are associated with depression [11]. In non-pregnancy studies, disrupted production and function of neurotransmitters, as well as the altered gut microbiome, have been identified as potential mechanisms linking nutrition deficiency to mood disorders [58], although these mechanisms require further research among pregnant women. Additionally, high consumption of processed carbohydrates, indicative of poor diet quality, can increase the risk of depression and anxiety through rapid changes in blood glucose levels [59,60]. Inflammation may also play a role in the association between poor diet quality and depression. For instance, a high-fat diet has been linked to inflammation (evidenced by increased levels of plasma cytokines and chemokines), leading to major mood disorders in mice [61]. A Western diet, characterized by high consumption of saturated fat and refined sugars, has been associated with increased rates of inflammation, depression, and cognitive impairment [62,63]. Possible mechanisms underlying the association between inflammation and mood disorders are direct effects of pro-inflammatory cytokines on monoamine levels, hypothalamic–pituitary–adrenal axis dysregulation, abnormal activation of microglial cells, impaired neuroplasticity, and structural and functional changes in the brain [64].

Both the HEI-2015 and ADII were associated with prenatal depressive symptoms. Compared to HEI-2015, ADII encompasses a weighted score of nutrients and dietary components that have demonstrated an association with biological markers of inflammation, including omega-3 polyunsaturated fatty acids, fiber, individual vitamins and minerals, caffeine, ethanol, trans fatty acids, and saturated fatty acids [24]. Thus, the current finding that ADII and prenatal depressive symptoms are correlated supports the association between inflammation and depression in the general population (see review articles, [65,66,67,68]) and extends the understanding of this link to diet and prenatal depression. Furthermore, the association between higher adherence to dietary guidelines and fewer prenatal depressive symptoms may be, in part, explained by other pathways not related to inflammation. For example, poor diet quality is associated with an increased risk of hyperglycemia and gestational diabetes in pregnancy [69]. In turn, gestational hyperglycemia has been associated with higher odds of prenatal depressive symptoms [70]. In our relatively small cohort, the effect size for the association between HEI-2015 and EPDS score holds practical significance when it is considered that, on a 2000 kcal diet, a 20-unit increase in the HEI-2015 can be practically achieved through, for example, adding 3 oz of whole grains to one’s daily diet (if the starting point is no whole grain intake) and reducing sodium intake to equal to or less than 2.2 g [71]. By making a significant impact on overall diet quality (i.e., 20-unit HEI-2015 score increase), our results suggest that such simple dietary changes could reduce the EPDS score by a total of four points. A four-point reduction in EPDS is recommended as the minimal clinically important difference [72,73]. Thus, progressive improvements in the maternal diet, in alignment with dietary guidelines, could achieve clinically meaningful effects on maternal mental well-being [74,75,76], with potential beneficial impacts on neonatal outcomes [75,77]. However, further research with larger sample sizes would be required to ascertain whether this effect size between HEI-2015 and EPDS score can be replicated.

Interestingly, the association between a pro-inflammatory diet and increased prenatal depressive symptoms was significant among pregnant women with worse economic well-being but not those with better economic well-being. Worse economic well-being might predispose pregnant women to a suboptimal health state that exacerbates the impact of a proinflammatory diet on prenatal depression. Among a nationally representative US adult sample, lower income was associated with higher concentrations of IL-6, C-reactive protein, and fibrinogen, which are indicative of poor health and disease risk [78]. Pregnant women living close to the poverty line might experience food insecurity [79], live in food deserts [80], have higher rates of illness [81] and health-damaging behaviors [82], as well as a lack of psychosocial protective factors such as social support [83], which are all crucial risk factors to worse overall health. In the current sample, Hispanic women were disproportionally affected by poor economic well-being. Previous research has also reported that within California, Hispanic women tend to underuse mental health services and underreport their depression symptoms [84]. As a result, this behavioral pattern contributes to an adverse state of overall health and well-being. Primary prevention targeting nutritional knowledge and anti-inflammatory dietary practices might be particularly helpful in promoting mental health among Hispanic pregnant women.

Several limitations should be considered when interpreting the results. First, from the standpoint of nutritional psychiatry, this study examined diet quality as a predictor variable for prenatal depression. However, bidirectional correlations may exist in the association between a pro-inflammatory state and depression [85]. Moreover, our cross-sectional data cannot establish a causal relationship between dietary intake and prenatal depressive symptoms. Second, individuals with a current clinical diagnosis of depression were not eligible for the studies. Therefore, it remains unknown whether the observed associations would hold true among women with clinical prenatal depression. Third, although we intentionally did not recruit pregnant women to the study with known substance abuse, we cannot guarantee that all participants in the research were free from substance abuse, which could represent an unknown confounder. Furthermore, even though our findings were expected based on existing literature, it is critical to replicate the results with a larger sample size. Lastly, dietary intake data collected through ASA24 may be influenced by social desirability and recall bias.

## 5. Conclusions

A higher-quality maternal diet, reflecting both an anti-inflammatory potential and adherence to dietary recommendations, is associated with fewer prenatal depressive symptoms. This study further provides novel and preliminary evidence that pregnant women with worse economic well-being may be more vulnerable to prenatal depressive symptoms when consuming a pro-inflammatory diet (i.e., a diet rich in saturated fat and sugar and with low consumption of fiber and micronutrient-rich foods).

Promoting a healthy diet in line with dietary guidelines might enhance the mental well-being of pregnant women. Implementing strategies to improve nutrition literacy and address dietary concerns during prenatal care could prove effective in promoting overall health among expectant mothers. Moreover, the moderating effect of economic well-being supports the notion that the relationship between diet and mental health can vary across different demographic subgroups. By acknowledging and thoroughly examining these variations, researchers can avoid making misleading claims rooted in oversimplified assumptions about the connection between diet and prenatal mental health. Future research should investigate the mechanism underlying the moderating effect of economic well-being on this relationship. If the results can be replicated, they may point towards the need for an accessible, individualized approach to prenatal depression prevention through intervention programs that utilize healthy nutrition and provide economic support. Educating pregnant women about the importance of a healthy, anti-inflammatory diet and facilitating improved prenatal nutrition through targeted nutrition support programs, especially among underserved and economically disadvantaged communities, may better support maternal mental health during pregnancy. 

## Figures and Tables

**Table 1:** Descriptive statistics of participant characteristics and main study variables of the total study cohort and stratified by study of enrollment and by ethnicity.

	Overall	Study 1 (*n* = 17)	Study 2 (*n* = 26)		Non-Hispanic (*n* = 12)	Hispanic (*n* = 31)	
	*M* (*SD*) or *n* (%)	*M* (*SD*) or *n* (%)	*M* (*SD*) or *n* (%)	*p*	*M* (*SD*) or *n* (%)	*M* (*SD*) or *n* (%)	*p*
Participant Characteristics:							
Age	28.84 (5.72)	29.41 (5.21)	28.46 (6.10)	0.60	32.75 (4.31)	27.32 (5.52)	0.004
Gestational age	130.70 (20.98)	116.47 (26.36)	140.00 (8.19)	0.002	128.17 (26.35)	131.68 (18.93)	0.68
Pre-pregnancy BMI	31.07 (4.97)	30.86 (6.35)	31.21 (3.96)	0.83	28.99 (4.89)	31.88 (4.84)	0.09
Ethnicity				0.38			–
Non-Hispanic	12 (27.9)	6 (35.3)	6 (23.1)		–	–	–
Hispanic	31 (72.1)	11 (64.7)	20 (76.9)		–	–	–
Main Study Variables:							
ADII	0.03 (2.91)	0.07 (2.25)	0.00 (3.39)	0.95	0.05 (2.46)	0.02 (3.12)	0.98
HEI-2015	60.63 (13.37)	64.84 (11.18)	57.39 (14.25)	0.08	64.41 (14.87)	59.15 (12.71)	0.27
Economic well-being	3.15 (3.26)	2.88 (2.92)	3.37 (3.57)	0.65	5.05 (3.65)	2.38 (2.81)	0.02
EPDS score	7.07 (4.19)	7.18 (4.61)	7.00 (3.99)	0.90	7.00 (3.16)	7.10 (4.58)	0.95

Notes. BMI = body mass index; ADII = Alternative Dietary Inflammatory Index; HEI-2015 = Healthy Eating Index-2015; EDPS = Edinburgh Postnatal Depression Scale.

**Table 2:** Bivariate correlation between main study variables and participant characteristics.

	1.	2.	3.	4.	5.	6.
1. ADII						
2. HEI-2015	−0.65 ***					
3. Income-to-poverty ratio	−0.29	0.06				
4. EDPS score	0.36 *	−0.42 **	0.22			
5. Age	−0.62 ***	0.44 **	0.39 *	−0.15		
6. GA	0.02	−0.27	0.15	0.05	−0.00	
7. Pre-pregnancy BMI	0.02	0.03	−0.30	−0.20	−0.14	−0.13

Notes. ADII = Alternative Dietary Inflammatory Index; HEI-2015 = Healthy Eating Index-2015, EDPS = Edinburgh Postnatal Depression Scale; GA = gestational age; BMI = body mass index. * *p* < 0.05, ** *p* < 0.01, *** *p* < 0.001.

**Table 3:** Hierarchical regression models predicting prenatal depressive symptoms.

**Model 1**					
	***b***	**Beta**	***p***	**95% CI**	
Step 1					*R*^2^ = 0.08; _adj_.*R*^2^ = −0.01; *F*(3, 30) = 0.85, *p* = 0.48
Age	−0.16	−0.20	0.26	[−0.44, 0.12]	
Gestational age	0.00	0.01	0.97	[−0.07, 0.07]	
Pre-pregnancy BMI	−0.19	−0.23	0.22	[−0.50, 0.12]	
Step 2					*R*^2^ = 0.23; _adj_.*R^2^* = 0.09; *F*_change_(2, 28) = 2.72, *p* = 0.08
ADII	0.60	0.40	0.06	[−0.03, 1.23]	
Economic well-being	0.36	0.28	0.15	[−0.14, 0.87]	
Step 3					*R*^2^ = 0.36; _adj_.*R*^2^ = 0.22; *F*_change_(1, 27) = 5.75, *p* = 0.02
ADII × Economic well-being	−0.18	−0.52	0.02	[−0.33, −0.03]	
	Overall *R*^2^ = 0.36; _adj_.*R*^2^ = 0.22; *F*(6, 27) = 2.57, *p* = 0.04				
**Model 2**					
	***b***	**Beta**	***p***	**95% CI**	
Step 1					*R*^2^ = 0.08; _adj_.*R*^2^ = −0.01; *F*(3, 30) = 0.85, *p* = 0.48
Age	−0.16	−0.20	0.26	[−0.44, 0.12]	
Gestational age	0.00	0.01	0.97	[−0.07, 0.07]	
Pre-pregnancy BMI	−0.19	−0.23	0.22	[−0.50, 0.12]	
Step 2					*R*^2^ = 0.32; _adj_.*R*^2^ = 0.20; *F*_change_(2, 28) = 4.92, *p* = 0.02
HEI-2015	−0.18	−0.53	0.01	[−0.30, −0.05]	
Economic well-being	0.31	0.24	0.19	[−0.16, 0.78]	
Step 3					*R*^2^ = 0.34; _adj_.*R*^2^ = 0.20; *F*_change_(1, 27) = 1.00, *p* = 0.33
HEI-2015 × Economic well-being	0.02	0.16	0.33	[−0.02, 0.06]	
	Overall *R*^2^ = 0.34; _adj_.*R*^2^ = 0.20; *F*(6, 27) = 2.34, *p* = 0.06				

Notes. ADII = Alternative Dietary Inflammatory Index; HEI-2015 = Healthy Eating Index-2015, EDPS = Edinburgh Postnatal Depression Scale; BMI = body mass index.

## Data Availability

The data presented in this study are available on request from the corresponding author. The data are not publicly available due to ethical restrictions.
